# Symptom Clusters in Survivorship and Their Impact on Ability to Work among Cancer Survivors

**DOI:** 10.3390/cancers15215119

**Published:** 2023-10-24

**Authors:** Joanna E. Fardell, Sim Yee (Cindy) Tan, Kim Kerin-Ayres, Haryana M. Dhillon, Janette L. Vardy

**Affiliations:** 1 UNSW Medicine & Health, School of Clinical Medicine, UNSW Sydney, Sydney 1466, Australia; j.fardell@unsw.edu.au; 2 Western Sydney Youth Cancer Service, Westmead Hospital, Sydney 2145, Australia; 3 Sydney Medical School, Faculty of Medicine and Health, University of Sydney, Sydney 2006, Australia; sim.tan@sydney.edu.au; 4 Concord Cancer Centre, Concord Repatriation General Hospital, Sydney 2139, Australia; kim.kerinayres@health.nsw.gov.au; 5 Psycho-Oncology Cooperative Research Group, School of Psychology, Faculty of Science, University of Sydney, Sydney 2006, Australia; haryana.dhillo@sydney.edu.au

**Keywords:** cancer, work, survivor, symptoms, cognitive symptoms, anxiety, depression, pain, fatigue

## Abstract

**Simple Summary:**

Lasting impacts and symptoms of cancer treatment can affect survivors’ daily lives. We explored how symptoms and symptom clusters can impact the ability to work among 561 cancer survivors previously diagnosed with breast, colorectal, and haematological malignancies, who were on average 58 years old and 1.5 years since diagnosis. We found almost 35% of survivors reported limitations in their ability to work. We identified three main symptom clusters: pain, emotional, and cognitive symptoms. Survivors experiencing these symptom clusters were approximately 14–17% more likely to report limitations in their work ability. Older survivors and those with more advanced cancer stages were also more likely to experience limitations in their work ability. In conclusion, a significant number of cancer survivors struggle to work due to their symptoms after treatment. Out results suggest that by better understanding and managing these symptoms, survivors can have improved opportunities to participate in work after their cancer treatment.

**Abstract:**

Background: Cancer survivors often experience a range of symptoms after treatment which can impact their quality of life. Symptoms may cluster or co-occur. We aimed to investigate how symptoms and symptom clusters impact the ability to work among cancer survivors. Methods: We used symptom severity data and ability to work data routinely collected from cancer survivors attending a survivorship clinic after primary treatment with curative intent. We defined symptom clusters using single linkage and a threshold on the rescaled distances of <10. We then conducted a logistic regression to examine how symptoms and symptom clusters were related to the ability to work. Results: We analysed data from 561 cancer survivors, mean age 58 years and 1.5 years post diagnosis, with mixed diagnoses including breast (40.5%), colorectal (32.3%), and haematological cancers (15.3%). Limitations to work ability were reported by 34.9% of participants. Survivors experiencing pain, emotional, and cognitive symptom clusters were 14–17% more likely to report limitations in their ability to work. Older survivors and those with a higher stage disease were more likely to report limitations in their ability to work. Conclusion: A better understanding and management of symptom severity and symptom clusters may help the sizable proportion of cancer survivors experiencing symptoms to participate in work after treatment.

## 1. Introduction

Many people surviving cancer experience a range of symptoms and complications during and/or following their cancer treatment that may persist long-term and lead to poorer health outcomes. This can include compromised physical functioning and psychological well-being, and the development of chronic illnesses, all of which can impact an individual’s quality of life and survival [1,2,3,4,5].

Our own research in 385 early-stage cancer survivors attending their initial visit at the Sydney Cancer Survivorship Centre Clinic found that approximately 12 months after diagnosis, 56% of survivors reported five or more symptoms of at least moderate severity (>=4/10) [3]. The most commonly reported symptoms were fatigue (45%), difficulty sleeping (37%), pain (34%), sore hands or feet (30%), numbness or pins and needles (30%), difficulties with thinking skills (concentration and memory [27.5%]), and mood disturbance (anxiety [31%], depression [23%], and stress [45%]), with fear of cancer recurrence rated by a clinical psychologist based on their consultation as 62% [3]. A second study comparing the rates and severity of symptoms at the initial visit to their follow up visit, 12 months later, found some improvement in pain, fatigue, and energy, but more than 20% of survivors still had moderately severe fatigue, pain, or sleep disturbance, and 26%, particularly women, continued to report five or more symptoms of at least moderate severity [6]. Others have reported similar symptoms but at even higher rates five years after adjuvant treatment in Asian and Australian cancer survivors: fatigue (67%), loss of strength (62%), pain (62%), sleep disturbance (60%), and weight changes (58%) [7].

Similar to our finding of high rates of survivors with multiple symptoms, previous research, which has been mainly conducted in breast cancer survivors, has documented the co-occurrence of symptoms, also known as symptom clusters, in the years after treatment, particularly for sleep, fatigue, and mood disturbance [8]. Symptom clusters have been defined as consisting of two or more symptoms that are related to each other and are considered to be relatively stable over time [9,10]. Other symptom clusters among survivors include ongoing pain and symptoms associated with peripheral neuropathy, cognitive impacts, and reduced physical activity [11,12,13]. Primary cancer site, disease stage, or antitumor treatment have previously been found to predict the occurrence of symptom clusters [14], but these associations with demographic and clinical variables are not consistently found [15]. For example in a secondary analysis of 282 breast cancer patients receiving chemotherapy or radiotherapy, Kim et al. [15] found no association of treatment modality (chemotherapy vs. radiotherapy), age, employment status, disease stage, and comorbid condition.

The reasons for symptom clusters include a common aetiology or biological pathway, such as an inflammatory process and increased cytokines, immune processes, activation of the sympathetic nervous system, and activation of the hypothalamic-pituitary–adrenal axis [10,16]. However, attribution of a clear and single causal pathway is challenging due to variable symptom cluster inclusions across papers (e.g., fatigue, pain, and depression versus fatigue, pain, depression, and sleep disturbance) [10].

Reasons for not participating in work at pre-diagnosis levels are multifactorial [17,18]. Symptom burden can impact an individual’s ability to resume daily activities including returning to work or the ability to achieve expected outcomes at work (for example, completing tasks on time or to a level of previous proficiency). In addition to providing an income, working or returning to work is often regarded as an important milestone for cancer survivors as it is extrinsically linked to identity, self-esteem, and a sense of returning to normalcy for those of working age [17,19]. However, up to 40% of cancer survivors either do not return to work or do not go back to their pre-diagnosis level of employment [20]. A recent systematic review of 68 studies in cancer survivors who had completed primary cancer treatment found a trend for higher symptom burden to be associated with poorer work outcomes [21]. Fatigue, depression, and cognitive symptoms were found to be related to poorer work performance, and issues with body image and oral dysfunction were associated with a higher risk of unemployment [21].

Cognitive symptoms (also referred to as cognitive impairment throughout the literature) are likely to contribute to work outcomes due to the requirements to utilise cognitive skills at work (e.g., pay attention, complete tasks within an allotted time, remember to do tasks, plan, organise, prioritise and review tasks) [17,22,23]. In a sample of 68 breast cancer survivors who had returned to work, Von Ah et al. found perceived cognitive impairment was associated with the ability to work, while clinical and demographic factors were not. Similar results indicating a role for perceived cognitive impairment impacting markers of work productivity (e.g., time management) and engagement were also found [18,24].

Self-report of cognitive symptoms have consistently been associated with emotional symptoms of depression and anxiety, as well as fatigue [25,26]. For example, our analysis of population level data (N = 10,337, with 691 (6.7%) with a history of cancer) found moderate to high correlation between anxiety, depression and concentration difficulties (correlations ranged r = 0.52 for anxiety and concentration difficulties, to r = 0.81 for anxiety and depression), and approximately 9% of the population reported experiencing either concentration difficulties and depression or concentration difficulties and anxiety [27]. However, few studies have considered the co-occurrence of cognitive symptoms, such as attention and memory difficulties, in addition to other commonly co-occurring symptoms, such as depression, anxiety, and fatigue, and its consequent impact on the ability to work. Among a sample of 379 cancer survivors, Ehrenstein et al. [26] found self-reported cognitive symptom (memory and executive function) trajectories were related to work, fatigue, and depression. They found more cognitive symptoms which were stable over time to be associated with older age, longer time after diagnosis before returning to work, more quantitative work demands, and higher levels of depressive symptoms at baseline. However, they did not investigate the co-occurrence of cognitive symptoms, fatigue, and depression, and their compounding impact on work outcomes. Considering symptom clusters that include cognitive difficulties, their impact on the ability to work is critical, given (i) the likelihood of multiple symptoms being experienced due to cancer treatment well into survivorship and (ii) the compounding negative impact of multiple symptoms on quality of life and the ability to participate in work. Therefore, to address this gap in the literature, this study aimed to (i) identify symptom clusters based on severity of symptoms, and (ii) evaluate how these symptoms, including self-reported cognitive symptoms, were related to work ability among a sample of cancer survivors.

## 2. Methods

### 2.1. Participants

Cancer survivors attending the Sydney Cancer Survivorship Centre multidisciplinary team clinic between September 2013 (when the clinic opened) and October 2020, who provided returning to work data and consented for their data to be used in research, were eligible to participate in this research study. Eligibility for attending the Sydney Cancer Survivorship Centre has been published [27] and includes an invasive cancer diagnosis, completion of primary cancer treatment (generally including chemotherapy) for early-stage cancer, or occasionally those with stage IV who have had potentially curative treatment, such as resection of metastatic disease, and no evidence of a cancer recurrence. Exclusion criteria for attending the clinic include currently undergoing primary treatment, with the exceptions that patients with breast cancer may be receiving hormonal treatment and/or targeted therapy such as trastuzumab, and patients with haematological malignancy may be on maintenance treatment. Referrals for the Sydney Cancer Survivorship Centre are accepted from medical oncologists, surgeons, and radiation oncologists [28].

### 2.2. Measures

Participants completed questionnaires either on paper or via online survey using the REDCap survey function prior to attending the Sydney Cancer Survivorship Centre clinic. The questionnaires included a comprehensive assessment of patient self-reported symptoms, quality of life, distress, and lifestyle factors [28]. For this analysis, we focused on self-reported symptoms and the ability to work.

Participants’ demographics, including age and sex and clinical information, including diagnosis, stage, treatment received, and time since treatment, were obtained from the patient’s medical record.

Self-reported symptoms were selected from the Patient’s Disease and Treatment Assessment (PDTA) form [29]. The PDTA asks respondents to rate the severity of 47 symptoms and aspects of wellbeing; we also added a memory question. Symptoms are rated from 0–10, with higher scores indicating greater symptom severity, and the following anchors are provided: 0 indicating no trouble and 10 indicating the worst I can imagine. For this analysis, we focused on symptoms commonly experienced during survivorship based on our included cohort of survivors [3,6] and included the following: pain, fatigue, trouble sleeping, numbness or pins and needles, diarrhoea, anxiety, depression, trouble concentrating, and problems with memory. Additionally, these symptoms were selected to ensure the coverage of the commonly occurring symptom clusters grouped into physical symptoms or psychological symptoms [14,30].

To determine whether participants were able to work, we used a single item from the Functional Assessment of Cancer Therapy–General (FACT-G): “I am able to work (include work at home)” [31]. Responses are on a 5-point Likert scale, ranging from 0 “not at all” to 4 “very much”. We dichotomised work ability as “limited ability to work” if the participant responded 0 “not at all”, 1 “a little bit”, or 2 “somewhat”; work ability was categorised as “able to work” if the participant responded 3 “quite a bit” or 4 “very much”.

### 2.3. Statistical Analysis

We conducted all statistical analyses using IBM SPSS Statistics version 27 (IBM Corporation, Armonk, NY, USA). We described our sample using means and standard deviations for continuous variables and counts and proportions for categorical variables. We compared participants’ work ability on demographic and clinical factors using chi-square tests for categorical variables and ANOVA for continuous variables. Previous research comparing statistical methods for identifying symptom clusters has found consistency across a principal component analysis, exploratory factor analysis, and hierarchical cluster analysis [30]. To address aim 1 and identify symptom clusters, we conducted a hierarchical cluster analysis. We defined clusters using single linkage and a threshold on the rescaled distances <10, as previously recommended [30]. We operationalised symptom cluster scores as the mean of the constituent symptoms. To address aim 2, we ran two logistic regression models with ability to work as the dependent variable. In the first model, we entered demographic variables and individual symptoms. In the second model, we entered demographic variables and any symptom clusters we had previously identified and individual symptoms. Factors were considered significantly associated with ability to work when *p* < 0.05.

## 3. Results

**Figure 1:**
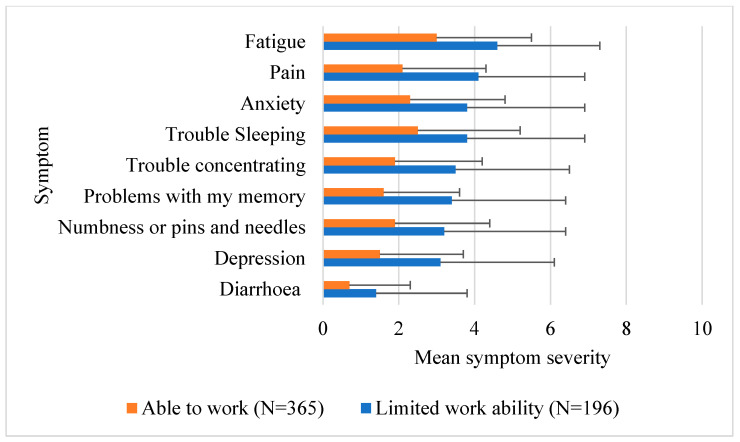
Mean symptom severity rating. Higher scores out of 10 indicate greater severity.

Ability to work data were available from 561 cancer survivors who attended the Sydney Cancer Survivorship Centre clinic. Table 1 provides the demographic characteristics of the population used for this analysis. Participants were on average 58 years old and 1.5 years post diagnosis. Most were female (69.2%), and the most common diagnosis was breast cancer (40.5%), followed by colorectal cancer (32.3%). Over a third (34.9%) of participants reported limitations in their ability to work. There was no difference between those who reported limited work ability and those able to work successfully based on clinical and demographic factors in univariable analysis. However, those with limited ability to work were more likely to report greater symptom severity than those who reported no limitation to working at the time of their initial visit (Figure 1, all *p* values ≤ 0.001). Fatigue did not differ significantly between those with limited work ability and those able to work (*p* = 0.177).

### 3.1. Symptom Clusters

**Figure 2:**
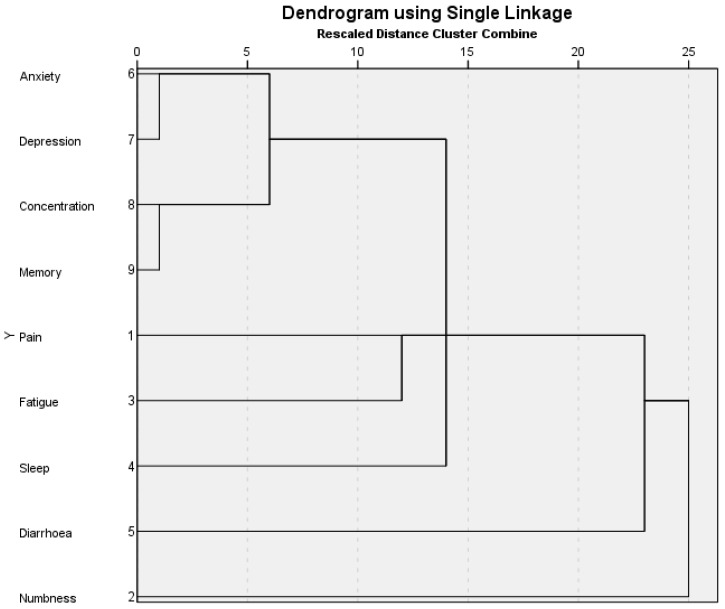
Dendrogram showing symptom clustering using single linkage as previously described [29].

We determined clusters at a rescaled distance of <10 (see Figure 2). At the lower threshold, clusters were identified as an emotional cluster (anxiety and depression), a cognitive cluster (attention and memory), or a combined emotional–cognitive symptom cluster.

### 3.2. Factors Associated with Returning to Work

In our first logistic regression model, we entered demographic and clinical variables into the model with individual symptoms to determine the factors associated with the ability to work after cancer (Table 2). The overall model fit was significant and accounted for 33.3% of the variance in self-reported work ability (χ^2^(17) = 133.25, *p* < 0.001). Of the demographic and clinical variables entered, we found older participants were less likely to be able to work (OR: 0.964, 95%CI: 0.944–0.985). Participants with Stage III and resected Stage IV disease were less likely to be able to work compared to those with Stage I disease (OR 0.385, 95%CI: 0.168–0.883). Symptoms of pain, fatigue, depression, and problems with memory were significantly associated with the ability to work, with those reporting more severe symptoms less likely to be able to work.

In our second logistic regression model (Table 3), we entered demographic and clinical variables into the model with the above identified symptom clusters and any remaining symptoms not included in a cluster to determine the factors associated with the ability to work after cancer. Again, the overall model fit was significant and accounted for 31.6% of the variance in self-reported ability to work (χ^2^(17) = 130.04, *p* < 0.001). Similar to the results of the first logistic regression model, older participants and those with Stage III and resected/treated Stage IV disease were less likely to be able to work. Participants reporting more severe pain, higher cognitive symptom cluster scores (i.e., more severe memory and attention symptoms on average), and those with higher emotion symptom cluster scores (i.e., more severe anxiety and depression symptoms on average) were less likely to be able to work.

## 4. Discussion

In our sample of 561 cancer survivors with mixed diagnoses who were attending a large survivorship multidisciplinary team clinic in Sydney, Australia, around 35% of survivors reported limitations in their ability to work. Reported symptom severity differed between those reporting limitations in their ability to work and those not reporting limitations in their ability to work after cancer. The factors that were significantly associated with increased odds of being able to work included younger age, early-stage disease, and less severe pain, emotional, and cognitive symptoms.

Our results are consistent with those of other studies showing impacts on work engagement and participation among cancer survivors [17,18,21,24,32]. Previous research has documented an associated impact on financial well-being, with survivors not able to work reporting reduced earnings [14,22,32]. The flow-on effect to overall quality of life is likely complex and age-dependent. Cancer survivors may reduce work hours or start retirement earlier than otherwise planned due to positive (i.e., cancer has caused a refocus on personal values such as spending more time with loved ones) and negative (i.e., the lasting symptoms of cancer treatment prohibit work and impact overall quality of life) impacts of cancer [17]. Our results do not tease apart these bidirectional impacts and future research is warranted. However, the average age of our sample was below the Australian retirement age (>65 years of age), and our results may reflect changed work plans, further highlighting the importance of assessing for lasting symptom impacts after cancer treatment is finished.

Similar to previous research, we found emotional or psychological symptoms of anxiety and depression clustered together, and cognitive symptoms of attention and difficulties with memory were clustered together [30,33]. Our results are important in highlighting that the ability to work after cancer is impacted by multiple symptoms, in addition to survivor age and disease stage. While these symptoms are self-reported, the presence of these symptoms was associated with returning to work. These results may reflect impacts on the capacity to fulfil work duties as highlighted by others [34]. In a sample of 1562 people with mixed diagnoses of advanced cancer, both on and off treatment, four symptom clusters were reliably identified using different statistical methods and included tense–worry–irritable–depressed (emotional cluster), fatigue–pain, nausea–vomiting, and concentration–memory (cognitive cluster) [30]. This study also found the emotional cluster was the strongest predictor of overall quality of life, in contrast to other identified clusters [30]. As cognitive symptoms, depression, and fatigue may all be relatively stable over time [26], this further highlights the importance of addressing psychosocial aspects in symptom cluster management [30] to help improve work outcomes for cancer survivors.

## 5. Implications for Cancer Survivors and Clinical Practice

We found older survivors and those with a higher stage of disease were at particular risk of not achieving their work goals and therefore may also be at a potentially increased risk of financial toxicity. Considering symptom presence (particularly pain and cognitive and emotional symptoms), severity, and any potential clustering or compounding effects during survivorship clinical encounters, particularly for older survivors and those with a higher stage disease, may be particularly helpful to support survivors in achieving their work-related goals. Although treating a symptom in isolation may miss the full impact on overall quality of life during survivorship, conversely, focusing on reducing the severity of one symptom may reduce or prevent escalation of the other symptoms [35]. As such, it is recommended that clinicians screen for the presence of multiple symptoms and determine if a sentinel symptom exists within a symptom cluster [10], as this may be amenable to an evidence-based intervention.

A review of previous intervention research for symptom clusters has found interventions often targeted one symptom only [10], though some studies have reported improvements in symptoms, in addition to the primary symptom targeted [36]. In a pilot randomised controlled trial with 86 patients with advanced lung, prostate, colorectal, or gynaecologic cancers receiving treatment, a two week audio-recorded and delivered training program of twelve relaxation, imagery, or distraction exercises reduced the severity of the pain, fatigue, and sleep disturbance symptom cluster at two weeks post intervention, compared to a waitlist control group [35]. However, no impact was observed on symptom interference with daily life [35].

## 6. Strengths and Limitations

Previous research has largely focused on breast cancer survivors, and a strength of our study is the inclusion of a large sample of survivors with mixed cancer diagnoses and stages; however, there are several limitations worth noting. We used a single item measure of the ability to work and the experience of symptoms. While these questions are used in current clinical encounters and serve as a reasonable screen of symptoms [37] and the ability to work, research studies employing a more comprehensive assessment of the presence of multiple symptoms and their severity, in addition to validated measures of the ability to work, are warranted [37]. To address this gap clinically, at the Sydney Cancer Survivorship Centre clinic, we have recently started collecting more information on patients’ work status, changes in their working plans since their cancer diagnosis, and their ability to work after cancer.

## 7. Conclusions

We found that almost 35% of cancer survivors attending a survivorship clinic reported limitations in their ability to work and that this was associated with age, stage of disease, and the presence of multiple symptoms including pain and emotional and cognitive symptoms. A better understanding of symptom severity and clusters, and reducing symptom burden after cancer treatment, may support survivors to better engage in work. Future research on work engagement and the ability of cancer survivors to work should seek to further explore and address the whole person context, including symptom burden and benefits and barriers to work after cancer, to better understand and support cancer survivors in achieving their work goals.

## Figures and Tables

**Table 1:** Participant demographics and symptoms for the whole sample and whether participants were able to work or were limited in their ability to work ^g^.

	Total Sample (*n* = 561)	Limited Work Ability (*n* = 196)	Able to Work (*n* = 365)
Age (SD)	58.0 (13.4)	60.5 (12.8)	56.6 (13.6)
Range	18–91		
Female sex	338 (69.2%)	140 (31.1%)	248 (63.9%)
Years since diagnosis (SD)	1.5 (2.0)	1.5 (1.6)	1.5 (2.1)
	Median 1.0		
75th Percentile	1.0		
Tumour type			
Breast	227 (40.5%)	77 (33.9%)	150 (66.1%)
Colorectal	181 (32.3%)	68 (37.6%)	113 (62.4%)
Haematological	86 (15.3%)	28 (32.6%)	58 (67.4%)
Other ^a^	67 (11.9%)	23 (34.3%)	44 (65.7%)
Stage ^b^			
I	83 (14.8%)	23 (27.7%)	60 (72.3%)
II	180 (32.1%)	59 (32.8%)	121 (67.2%)
III and resected IV ^c^	279 (49.7%)	106 (38.0%)	173 (62.0%)
Treatment received			
Surgery ^d^	469 (83.6%)	166 (35.4%)	303 (64.6%)
Chemotherapy ^e^	478 (85.2%)	165 (34.5%)	313 (65.5%)
Radiotherapy ^f^	235 (41.9%)	79 (33.6%)	156 (66.4%)

Notes: ^a^ Other cancer types includes lung (*n* = 20), upper gastrointestinal (*n* = 36), and other diagnoses (*n* = 11); ^b^ missing 19; ^c^ *n* = 40 with Stage IV disease at diagnosis; ^d^ missing 11; ^e^ missing 8; ^f^ missing 12; ^g^ able to work was defined as having responded “quite a bit” or “very much” to the item “I am able to work (including work at home)”; limited work ability was defined as those having responded “not at all”, “a little bit” or “somewhat”.

**Table 2:** Logistic regression with work ability as the dependent variable and clinical and demographic variables and symptoms as independent variables.

	OR	95% C.I Lower	95% C.I Upper	*p*-Value
Participant age at survey completion	0.964	0.944	0.985	0.001
Sex	0.841	0.471	1.502	0.558
Tumour type				
Breast (reference)	-	-	-	-
Colorectal	0.918	0.432	1.951	0.823
Haematological	0.264	0.031	2.226	0.221
Other	0.989	0.403	2.431	0.981
Stage				
Stage I (reference)	-	-	-	-
Stage II	0.578	0.266	1.256	0.166
Stage III and resected Stage IV	0.385	0.168	0.883	0.024
Treatment				
Surgery	0.306	0.042	2.212	0.241
Chemotherapy	0.875	0.413	1.854	0.728
Radiotherapy	1.125	0.627	2.021	0.693
Symptoms				
Pain (all and anywhere)	0.833	0.748	0.928	0.001
Numbness or pins and needles	0.934	0.858	1.016	0.111
Fatigue (tiredness)	0.889	0.786	1.006	0.062
Trouble sleeping	1.037	0.933	1.152	0.501
Diarrhoea	0.969	0.857	1.094	0.609
Anxiety (feeling worried)	0.977	0.843	1.132	0.759
Depression (feeling sad)	0.865	0.748	0.999	0.049
Trouble concentrating	1.097	0.947	1.271	0.217
Problems with memory	0.798	0.702	0.907	0.001

**Table 3:** Logistic regression with work ability as the dependent variable and clinical and demographic variables and symptom clusters identified using a rescaled distance of <10 as independent variables.

	OR	95% C.I Lower	95% C.I Upper	*p*-Value
Participant age at survey completion	0.962	0.943	0.982	0.000
Sex	0.777	0.442	1.367	0.382
Tumour Type				
Breast (reference)	-	-	-	-
Colorectal	1.069	0.516	2.214	0.858
Haematological	0.480	0.072	3.185	0.447
Other	1.177	0.490	2.828	0.715
Stage				
Stage I (reference)	-	-	-	-
Stage II	0.491	0.231	1.045	0.065
Stage III and resected Stage IV	0.331	0.148	0.740	0.007
Treatment				
Surgery	0.468	0.080	2.736	0.400
Chemotherapy	0.981	0.482	1.997	0.958
Radiotherapy	1.108	0.631	1.947	0.721
Symptoms				
Pain (all and anywhere)	0.830	0.747	0.921	0.000
Numbness or pins and needles	0.927	0.855	1.005	0.068
Fatigue (tiredness)	0.909	0.807	1.024	0.117
Trouble sleeping	1.055	0.954	1.166	0.295
Diarrhoea	0.969	0.861	1.091	0.603
Emotional symptom cluster	0.858	0.764	0.965	0.010
Cognitive symptom cluster	0.864	0.765	0.975	0.018

## Data Availability

Data are available on reasonable request from the author.
